# Sustained release of a highly specific GSK3β inhibitor SB216763 in the PCL scaffold creates an osteogenic niche for osteogenesis, anti-adipogenesis, and potential angiogenesis

**DOI:** 10.3389/fbioe.2023.1215233

**Published:** 2023-07-28

**Authors:** Weimin Gong, Molin Li, Lizhou Zhao, Pengtao Wang, Xiaofang Wang, Bo Wang, Xing Liu, Xiaolin Tu

**Affiliations:** ^1^ Laboratory of Skeletal Development and Regeneration, Institute of Life Sciences, Chongqing Medical University, Chongqing, China; ^2^ Department of Orthopedics, Ministry of Education Key Laboratory of Child Development and Disorders, National Clinical Research Center for Child Health and Disorders, Children’s Hospital of Chongqing Medical University, Chongqing, China

**Keywords:** SB216763, Wnt signaling, osteogenesis, adipogenesis, angiogenesis, PCL and cell integrated 3D scaffold, sustained release

## Abstract

The safe and effective use of Wnt signaling is a hot topic in developing osteogenic drugs. SB216763 (S33) is a widely used highly specific GSK3β inhibitor. Here, we show that S33 initiates canonical Wnt signaling by inhibiting GSK3β activity in the bone marrow stromal cell line ST2 and increases osteoblast marker alkaline phosphatase activity, osteoblast marker gene expression including *Alpl*, *Col1α1*, and *Runx2*, promoting osteogenic differentiation and mineralization of ST2 cells. In addition, S33 suppressed the expression of adipogenic transcription factors *Pparg* and *Cebpa* in ST2 cells to suppress adipogenesis. ICRT-14, a specific transcriptional inhibitor of Wnt signaling, reversed the effects of S33 on the differentiation of ST2 cells. S33 also increased the expression of osteoclast cytokines *RANKL* and *Opg* but decreased the *RANKL/Opg* ratio and had the potential to inhibit osteoclast differentiation. In addition, we printed the PSCI3D (polycaprolactone, S33, cell-integrated 3D) scaffolds using a newly established integrated 3D printing system for hard materials and cells. S33 sustained release in the hydrogel of the scaffold with 25.4% release on day 1% and 81.7% release over 7 days. Cells in the scaffolds had good cell viability. The ratio of live/dead cells remained above 94% for 7 days, while the cells in the scaffolds proliferated linearly, and the proliferative activity of the PSCI3D scaffold group increased 1.4-fold and 1.7-fold on days 4 and 7, respectively. Similarly, in PSCI3D scaffolds, osteogenic differentiation of st2 cells was increased. The alkaline phosphatase activity increased 1.4- and 4.0-fold on days 7 and 14, respectively, and mineralization increased 1.7-fold at 21 days. In addition, PSCI3D conditioned medium promoted migration and tubulogenesis of HUVECs, and S33 upregulated the expression of *Vegfa*, a key factor in angiogenesis. In conclusion, our study suggests that S33 functions in osteogenesis, anti-adipogenesis, and potential inhibition of osteoclast differentiation. And the sustained release of S33 in PSCI3D scaffolds creates a safe osteogenic niche, which promotes cell proliferation, osteogenesis, and angiogenesis and has application prospects.

## Introduction

More effective osteogenic drugs with fewer side effects remain a research hotspot. Teriparatide (PTH), abalopathide (PTHrP), and romosozumab (sclerotin antibody) are anabolic drugs approved for the treatment of osteoporosis. PTH and PTHrP act through PTH receptors to regulate the fate of BMSCs towards osteoblasts or adipocytes in bone (Fan et al., 2017) and to promote angiogenesis as well (Cohn-Schwartz et al., 2022), but PTH and PTHrP should not be used for a long time because of the risk of inducing osteosarcoma (Neer et al., 2001). Sclerostin antibodies activate the Wnt/β-catenin pathway to promote bone formation and reduce bone resorption (McClung et al., 2014). However, sclerotin antibodies may lead to vascular calcification (Golledge and Thanigaimani, 2022), increasing the risk of severe cardiovascular disease (Saag et al., 2017).

Targeting Wnt signals may be an effective way for drug development (Baron and Kneissel, 2013). The Wnt signaling is associated with cell proliferation, differentiation, polarization, and migration (Wodarz and Nusse, 1998). *ß*-catenin levels are critical regulators of Wnt signaling. They can be regulated by a ubiquitin-dependent proteolysis system when the Wnt ligand binds to the receptor Frizzled and the co-receptor low-density lipoprotein (Lrp) 5/6 to form a complex, which inhibits the effect of the *ß*-catenin destruction complex, resulting in reduced *ß*-catenin phosphorylation to stabilize *ß*-catenin in the cytoplasm. Increased *ß*-catenin enters the nucleus to cooperate with the transcription factor Tcf/Lef to activate the transcription of Wnt target genes (Nusse and Clevers, 2017). GSK3β is a member of the complex, and inhibiting its activity can disable the *ß*-catenin destruction complex, effectively activating Wnt/β-catenin signaling. This makes GSK3β a target for drug development (MacDonald et al., 2009).

SB216763 (S33) is a widely used GSK3β inhibitor with more than 500 articles published as searched by title/abstract in PubMed. S33 inhibits GSK3β activity by competitively inhibiting ATP activity (Coghlan et al., 2000). S33 is highly selective and does not affect the activity of 24 other serine/threonine and tyrosine protein kinases under complete inhibition of GSK3 activity. S33 has been used in many disease-related studies, such as neurogastrointestinal disorders (Zhang et al., 2017), denervated muscle atrophy (Weng et al., 2018), and bronchopulmonary dysplasia (Hummler et al., 2013), but its effects on mesenchymal stem cell differentiation and osteoporosis have not been reported.

In this study, we found that S33 initiates canonical Wnt signaling in the bone marrow stromal cell line ST2 and preosteoblastic cell line MC3T3-E1, promoting osteogenesis in a dose-dependent manner. S33 increases the activity of osteoblast marker alkaline phosphatase and the expression of osteoblast marker genes *Alpl*, *Col1α1*, and *Runx2*. In addition, S33 inhibits the transcription factor of adipogenesis *Pparg* and *Cebpα* to inhibit adipogenesis and potential osteoclast differentiation. These features make S33 a promising candidate drug suitable for drug development in preventing and treating elderly osteoporosis.

The materials in the orthopedic market are mostly metal, ceramic, polymer materials, etc., but the current gold standard for bone transplantation is still autologous bone transplantation (Herrmann et al., 2015). Because pure material grafts have low biological activity and lack bone regeneration ability, they are prone to loosening, graft failure, or inevitable revision outcomes 5—20 years after surgeries (Cha et al., 2019). Revision is a headache for orthopedic surgeons because it takes a lot of cost and time and impacts patients’ quality of life. Bone tissue engineering technology, especially 3D biological printing technology, has successfully achieved the personalized shape adaptation of tissue engineering scaffolds through live cell printing, creating a suitable biological microenvironment for cell adhesion, proliferation, and differentiation (Kang et al., 2022).

3D printing can solve the problem of shape matching, but the research of orthopedic materials failed to be considered from the perspective of bone developmental biology, resulting in effective bone regeneration.1 Therefore, the lack of biological activity is still a bottleneck problem. To address the biological activity of orthopedic materials and achieve a breakthrough in the “0″status of grafts combined with hard materials and stem cells in the current graft market, an integrated 3D tissue and organ printer (ITOP, in 2016) prints structures containing supportive hard materials and stem cells, forming vascularized bone in the body (Kang et al., 2016). This work has been featured as an industry milestone and a direction for future medical applications in journals with high-impact factors, such as Nature Reviews (Lieben, 2016; Malda and Groll, 2016; Pashuck and Stevens, 2016). However, 7 years later, there has been no breakthrough in the construction of biomimetic bones, and such research is still limited to 3D printing for the production of vascularized bones (Wang S. et al., 2022). There are few reports about other cells or tissues forming in orthopedic materials. The reason could be the lack of an osteogenic microenvironment with developmental function (Grosso et al., 2017).

Endowing hard material osteogenic niches is an improved approach to addressing bottlenecks (Liu et al., 2022; Wang et al., 2023). In this study, we tried to sustainably release S33 in hard material (PSCI3D) scaffolds, creating an osteogenic niche that completely solves cell survival and growth in the scaffolds, with a cell survival rate as high as 94.0%. Within 7 days, the proliferative activity of the cells increases linearly, while S33 is slowly released by 81.7%; however, amazingly, the scaffolds keep better osteoblast differentiation and mineralization after 7 days. Thus, the sustained release of S33 creates a safe osteogenic niche, which bionomically reconstructs functional bone with osteogenesis, angiogenesis, anti-adipogenesis, and potentially anti-osteoclastogenesis in the scaffold.

## Materials and methods

### Reagents and cells

Chemicals: S33 and ICRT-14 were purchased from MCE (Shanghai, China). Lyophilized Gelatin Methacryloyl (GelMA) and a photo-cross-linking agent (LAP) were obtained from Sunp Biotech (Beijing, China). Polycaprolactone (PCL) was obtained from Sigma (St Louis, MO, USA), while Trizol was purchased from Invrogen (Thermo Fisher Scientific, Carlsbad, CA, USA). Alizarin red S was obtained from Solarbao Biotechnology (Beijing, China), and Matrigel was purchased from Corning (Costar; Corning, NY, USA). Lipo 8000 and an enhanced chemiluminescence (ECL) reagent were obtained from Beyotime Biotechnology (Shanghai, China).

Assay kits: The BCIP/NBT alkaline phosphatase (AP) color-rendering kit, live/dead viability assay kit, alkaline phosphatase activity assay kit, cytoplasmic and nuclear protein extraction kit, and modified Oil Red O staining Kit and DAPI staining solution were all obtained from Beyotime Biotechnology (Shanghai, China). AG RNAex Pro Reagent and reverse transcription reaction kits were obtained from Accurate Biology (Hunan, China), while the CCK8 assay kit was purchased from MCE (Shanghai, China).

Antibodies: Wanlei (Shenyang, China) provided anti-β-catenin, anti-β-actin, and anti-lamin B antibodies, while Beyotime Biotechnology (Shanghai, China) supplied FITC-labeled goat anti-rabbit IgG (H + L) and HRP-labeled goat anti-rabbit IgG (H + L).

Plasmid: The Wnt signaling reporter construct and its control, TopFlash and FopFlash plasmids, were acquired from Addgene (Cambridge, MA, USA).

Cell culture reagents and cell lines: Gibco (Beijing, China) supplied *a*-MEM, DMEM, penicillin, and streptomycin, while Sigma (St. Louis, MO, USA) provided *ß*-glycerophosphate, ascorbic acid, dexamethasone, Indomethacin, and IBMX. Fetal bovine serum (FBS) was purchased from Biological Industries (Israel), and Wnt3a-expressing cells, control L cells, and human umbilical vein endothelial cells (HUVECs) were obtained from the American Type Culture Collection (ATCC, Manassas, VA, USA). Additionally, ST2 and MC3T3-E1 cells were acquired from Dr. Steve Teitelbaum.

### Cell culture and differentiation

ST2, Wnt3a, and control L cells were grown in a growth medium comprising 90% *a*-MEM, 10% FBS, 50U/mL penicillin, and 50 μg/mL streptomycin at 37°C and 5% CO_2_, with half of the medium being refreshed every 2 days. To induce osteogenic differentiation, cells were cultured in an osteogenic medium containing 10 mM *ß*-glycerophosphate and 50 μg/mL ascorbic acid, with medium replacement every 1–2 days. Adipogenic differentiation was induced by culturing the cells in an adipogenic medium consisting of 0.5 µM dexamethasone, 0.5 mM IBMX, and 60 µM Indomethacin in DMEM, with medium replacement every 3–4 days.

### Modulation of Wnt signaling

To activate and obstruct Wnt signaling, S33, and ICRT-14 were used, respectively. While S33 was employed at different concentrations to stimulate Wnt signaling, DMSO was the control. ICRT-14 was utilized to decrease the binding efficiency of *ß*-catenin to the N-terminal domain of T-cell factor 4 (TCF4) to stop *ß*-catenin activity (Gonsalves et al., 2011). And ST2 cells were split in each well of a 24-well plate at a density of 2 × 10^4^ cells and treated with S33 and ICRT-14 for 72 h to verify the specificity of Wnt signaling.

### Integrated 3D printing

As previously described in our earlier report (Wang P. et al., 2022), GelMA hydrogel was utilized to load both cells and S33. To prepare the GelMA solution, 0.5% (w/v) lithium phenyl-2, 4, 6-trimethylbenzoylphosphinate (LAP) photo-initiator was added to a mixture of 0.25 mL *a*-MEM and either 10 μM S33 or 5 × 10^5^ ST2 cells. Pre-melt at 95°C for 30 min to reach the optimal temperature for PCL printing; melted PCL was then extruded at 400 µm diameter with an interval of 1,100 µm and a printing speed of 2 mm/s. At 25°C, S33 or cell-loaded GelMA solution was printed between the PCL strips at a printing speed of 5 mm/s, with a beam diameter of 300 μm and an interval of 500 μm (Billiet et al., 2014). Once a layer was printed, the GelMA hydrogel was crosslinked under 405 nm blue light for 10s before printing resumed vertically for the next layer. After printing three layers, the PCL, S33, cell integrated 3D (PSCI3D) scaffold was successfully fabricated.

### Cell viability

To assess cell viability, we employed the same methods described in our previous report (Wang B. et al., 2022). Both live/dead assay kit and CCK8 proliferation activity assay were utilized for this purpose.

#### Live/dead assay

The live/dead cell assay was performed with a kit. Following culturing for 1, 4, and 7 days, the scaffold underwent washing with PBS and subsequent incubation in a mixture at room temperature. The mixture was prepared according to Calcein AM: PI: assay buffer = 1: 1: 1,000. Calcein AM for living cells, and PI for dead cells. The scaffold was then incubated for 0.5 h in a 37°C incubator before being imaged with a laser scanning confocal microscope (Nikon, Japan). Using ImageJ, the cell viability was quantified.

#### Cell proliferative activity

Cell proliferative activity was measured by the CCK8 kit. For the proliferation of ST2 cells, they were seeded in 96-well plates at a density of 2,000 cells per well and treated with varying concentrations of S33 for 24, 48, and 72 h. After treatment, 90 µL PBS and 10 µL CCK8 solution were added to each well and incubated at 37°C for 2 h. The absorbance at 450 nm was measured by a spectrophotometer (Thermo Fisher Scientific). For PSCI3D scaffolds, perform as above after 1, 4, and 7 days of incubation.

### Gene expression analysis

To analyze gene expression, total RNA was extracted from the cells using Trizol (Tu et al., 2012b). The cDNA was synthesized using AG RNAex Pro Reagent, and the cDNA was used as templates for quantitative real-time polymerase chain reaction (qPCR) to detect genes with primer sets (Tables 1, 2). The housekeeping gene glyceraldehyde-3-phosphate dehydrogenase (Gapdh) was used to normalize the mRNA expression levels, and the 2-Δ Ct method was applied (Tu et al., 2015).

**TABLE 1:** Sequences of primers used for RT-PCR (mouse).

Primer	Forward (5′-3′)	Reverse (5′-3′)
*Gapdh*	GCA​CAG​TCA​AGG​CCG​AGA​AT	GCC​TTC​TCC​ATG​GTG​GTG​AA
*beta-actin*	AGA​GGG​AAA​TCG​TGC​GTG​AC	CCA​TAC​CCA​AGA​AGG​AAG​GCT
*Lef1*	TAC​CCC​AGC​CAG​TGT​CAA​CA	TCC​ATG​ATA​GGC​TTG​ATG​ACT​TTC
*Axin2*	TGC​AGG​AGG​CGG​TAC​AGT​TC	GCT​GGA​AGT​GGT​AAA​GCA​GCT​T
*Alpl*	CAC​GGC​GTC​CAT​GAG​CAG​AAC	CAG​GCA​CAG​TGG​TCA​AGG​TTG​G
*Runx2*	CCGGTCTCCTTCCAGGAT	GGGAACTGCTGTGGCTTC
*Bglap*	CAGCGGCCCTGAGTCTGA	GCC​GGA​GTC​TGT​TCA​CTA​CCT​TA
*Col1a1*	GAC​AGG​CGA​ACA​AGG​TGA​CAG​CAG​AGG	CAG​GAG​AAC​CAG​GAG​AAC​CAG​GAG
*Ibsp*	CAG​AGG​AGG​CAA​GCG​TCA​CT	GCT​GTC​TGG​GTG​CCA​ACA​CT
*Pparg*	GCC​AAG​GTG​CTC​CAG​AAG​ATG​AC	GTG​AAG​GCT​CAT​GTC​TGT​CTC​TGT​C
*Cebpa*	GCCAAACTGAGACTCTTC	TGGCATCTCTGTGTCAAC
*RANKL*	CATGACGTTAAGCAACGG	AGGGAAGGGTTGGACA
*Opg*	ACG​GAC​AGC​TGG​CAC​ACC​AG	CTC​ACA​CAC​TCG​GTT​GTG​GG
*Vegfa*	AGA​AGG​AGG​AGG​GCA​GAA​TCA​TCA​C	GGGCACACAGGA TGGCTTGAAG

**TABLE 2:** Sequences of primers used for RT-PCR (human).

Primer	Forward (5′-3′)	Reverse (5′-3′)
*GAPDH*	GGA​GCG​AGA​TCC​CTC​CAA​AAT	GGC​TGT​TGT​CAT​ACT​TCT​CAT​GG
*VEGFA*	AGA​GGG​AAA​TCG​TGC​GTG​AC	CCA​TAC​CCA​AGA​AGG​AAG​GCT

### Osteogenic differentiation

#### Alkaline phosphatase staining

Alkaline Phosphatase Staining (AP staining) was performed according to a previously reported method (Tu et al., 2007). Following treatment with S33 for 3 days, ST2 cells were washed with PBS and fixed in 3.7% formaldehyde for 5 min. Next, washed thrice with PBS and subjected to staining using the BCIP/NBT alkaline phosphatase color development kit. For the PSCI3D scaffold, staining was carried out for 4 hours after culturing for 7 and 14 days. The plates and scaffolds were stained for 30 min and 4 h, respectively. Results were recorded using a digital camera.

#### AP biochemical activity assay

The cells were subjected to a previously reported assay to measure the alkaline phosphatase (AP) biochemical activity (Tu et al., 2007). Briefly, 0.3 mL of 10 mM Tris/HCl (pH 7.4) was added to each well, and the cells were scraped and sonicated three times for 10 s each. Centrifuged at 13,000 rpm for 3 min, the supernatant was collected and analyzed using an AP detection kit, following the manufacturer’s instructions.

#### Mineralization assay (Alizarin Red S staining)

ST2 cells and PSCI3D scaffold were cultured in a growth medium for 3 days and 7 days, respectively. The medium was then changed to osteogenic medium for both cell types and cultured for 14 days. To analyze matrix mineralization, cells, and scaffold were stained with 0.4% Alizarin Red S for 0.5 h and imaged under a microscope. After that, they were washed with PBS at room temperature and destained with 10% cetylpyridinium chloride for 1 h. The quantitation of mineralization was performed by measuring the absorbance of the washing solution at 562 nm.

### Plasmid transfection and reporter gene activity assay

The experimental protocol followed previous reports (Luo et al., 2022). ST2 cells were cultured into 24-well plates and transfected with TopFlash and FopFlash plasmids using Lipo8000 for 24 h. Transfected ST2 cells were continued to be treated with S33 for 48 h. Luciferase activity was measured using a dual luciferase reporter analysis system, and the results were presented as normalized values.

### Detection of changes of *ß*-catenin content

Immunofluorescence was used to detect *ß*-catenin in the treated cells, as previously reported (Liu et al., 2022). The cells were first treated with S33 or DMSO for 24 h, then washed with PBS and fixed with 4% paraformaldehyde. To allow for permeabilization, the cells were treated with PBS-T for 30 min, then blocked with 1% BSA for 0.5 h. Immunostaining was carried out using a rabbit polyclonal anti-mouse *ß*-catenin antibody (diluted 1:50) and FITC-labeled goat anti-rabbit IgG (H + L) secondary antibody (diluted 1:500). After three washes with PBS, the cells were incubated with DPAI (diluted 1:5000) for 5 min and washed again with PBS. Then, images were captured using a fluorescence microscope.

### Adipogenesis assay

#### Adipogenic differentiation

To induce adipogenic differentiation, the ST2 cells were cultured in an adipogenic medium (0.5 µM dexamethasone, 0.5 mM IBMX, 60 µM Indomethacin, and *a*-MEM was replaced by DMEM). Lipid droplets were observed microscopically after 7 days of stimulation with S33, followed by Oil Red O staining.

#### Oil Red O staining

ST2 cells were washed once with PBS. Then the cells were fixed with 3.7% formaldehyde for 5 min. ST2 cells were then washed three times with PBS at room temperature, stained with a modified Oil Red O staining kit per instruction, and photographed under a microscope.

### Western blotting

As we previously described (Tu et al., 2012a), the cytoplasmic and nuclear proteins were isolated from ST2 cells using a cytoplasmic and nuclear protein extraction kit. Samples containing 10 μg of protein were separated using a 10% SDS/PAGE gel and transferred onto a PVDF membrane (Millipore). Following blocking in 5% non-fat milk for 2 h, the membranes were incubated overnight at 4°C with the appropriate primary antibody: rabbit polyclonal anti-mouse *ß*-catenin antibody (diluted 1:1,000), mouse polyclonal anti-Lamin-B (diluted 1:1,000), or anti-β-actin (diluted 1:1,000) antibodies. After washing three times with TBS-T, the membranes were incubated with a secondary antibody, goat anti-rabbit IgG (H + L) (diluted 1:5000) for 1 h at room temperature. Following three additional washes with TBS-T, an enhanced chemiluminescence (ECL) reagent was used. The bands were measured by Bio-Rad XRS (Bio-Rad, Hercules, CA, US).

### S33 release experiment in scaffold

As reported (Wang et al., 2021), different concentrations of S33 were prepared ranging from 0.1 to 2 mg/mL, measuring the absorbance at 220 nm. A standard curve was then generated for absorbance values for S33 concentration. The PSCI3D scaffolds were placed in a 6-well plate, and added 4 mL of purified water. The plates were then incubated at 37°C. 1 mL of culture medium was measured for absorbance values at 220 nm and repeated at each time point. Then, the standard curve was used to obtain the change of S33 release in the PSCI3D scaffold over time. To maintain the volume, add 2 mL of fresh water back to the plate.

### Angiogenesis assay

#### PSCI3D scaffold conditioned medium preparation

After culturing the PSCI3D scaffold or DMSO control scaffold in a complete medium for 3 days, a conditioned medium (CM) was collected after that. Then they were stored at − 80°C until use. Wnt3a and the control L conditioned medium were collected in the same manner as previously reported (Vinyoles et al., 2014). Wnt3a-expressing cells and control L cells were cultured in complete medium for 3 days, and the conditioned medium (CM) was collected and stored at −80°C until use.

#### Cell migration assay

As previously reported (Liu et al., 2022), 1 × 10^5^ HUVECs were seeded in DMEM without FBS in the upper chamber. The lower chamber was filled with 700 μL DMEM containing 10% FBS and 300 μL CM, then placed at 37°C for 48 h. Migrated cells in the upper chamber were fixed with 3.7% formaldehyde for 5 min. Staining is then performed with a crystal violet stain for 1–2 min. Finally, stained cells were counted and quantified with ImageJ.

#### Tube formation assay

As we previously reported (Wang P. et al., 2022), HUVECs were seeded onto 24-well plates, which were pre-coated with 200 μL/well Matrigel at a density of 2 × 10^4^ per well and then incubated with 700 μL complete medium and 300 μL of PSCI3D scaffold conditioned medium for 6 h. The structures were observed and counted microscopically. Vascular tubular networks formed by HUVECs were quantified with ImageJ.

### Statistical analysis

GraphPad Prism 8.0.1 software was used for statistical analysis. Each experiment was independently repeated three times, and the results were presented as means ± SD (standard deviation). Differences between multiple groups were analyzed using One-Way ANOVA, while differences between groups split into two independent variables were analyzed using Two-Way ANOVA. Student’s t-test was used for analyzing differences between two comparable groups. A significant difference was defined as *p* < 0.05.

## Results

### S33 inactivates GSK3β to activate canonical Wnt signaling

**FIGURE 1:**
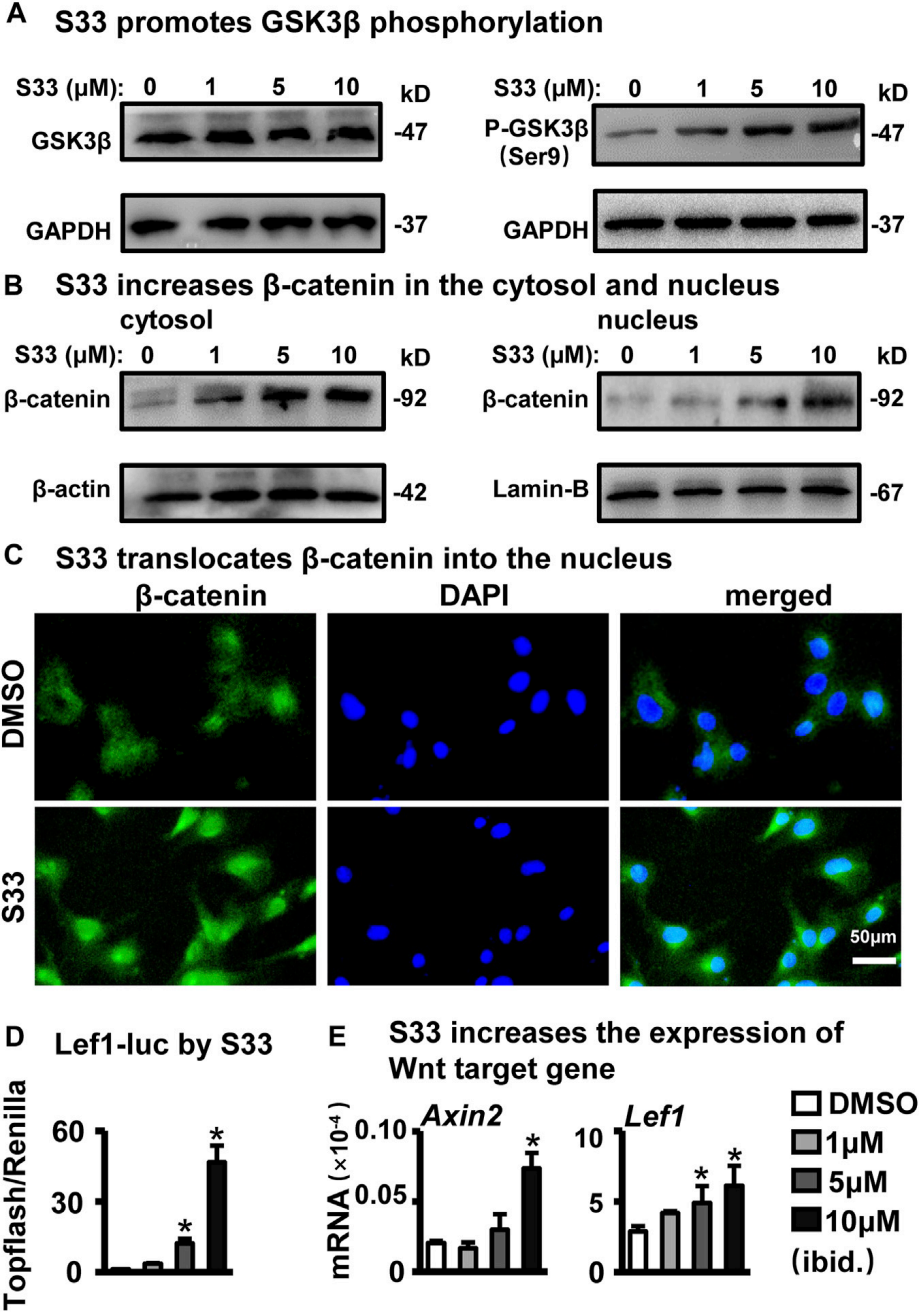
Effect of S33 on canonical Wnt signaling. **(A,B)** Western blotting detection of protein GSK3β, P-GSK3β, and *ß*-catenin expression in the cytoplasm and nucleus of ST2 cells treated with different concentrations of S33 **(C)** Immunofluorescence staining of *ß*-catenin in ST2 cells. Scale bar = 50 μm. **(D)** Luciferase activity of ST2 cells **(E)** Expression of Wnt targets genes. *, *p* < 0.05, compared with the DMSO group, n = 3.

As a highly selective GSK3β inhibitor, S33 can inhibit the activity of GSK3β without affecting the activity of other kinases (Zhu et al., 2019). Phosphorylation of GSK3β at SER9 leads to the inactivation of its enzymatic activity, a critical step in activating canonical Wnt signaling by inhibiting GSK3β. As shown in Figure 1A, s33 upregulated the phosphorylation level of SER9 of GSK3β in a dose-dependent manner in ST2 cells, whereas total GSK3β levels remained unchanged. Inactivation of GSK3β decreased the levels of phosphorylated *ß*-catenin and stabilized *ß*-catenin in the cytoplasm, and the stabilized *ß*-catenin entered the nucleus to activate Wnt signaling (MacDonald et al., 2009; Baron and Kneissel, 2013). The effect of S33 on intracellular *ß*-catenin levels was investigated by Western blotting. Compared with DMSO controls, *ß*-catenin protein content in cytosolic and nuclear fractions also increased progressively with increasing concentrations of S33 (Figure 1B).

To verify whether *ß*-catenin nuclear entry was increased, immunofluorescence staining by an anti-β-catenin antibody detected changes in *ß*-catenin levels in the nuclei of ST2 cells treated with 10 μM S33. Consistent with WB results, 10 μM S33 significantly promoted *ß*-catenin nuclear entry (Figure 1C).

Next, to determine the effect of S33 on canonical Wnt signaling transduction, we transfected ST2 cells with TopFlash (the synthetic *ß*-catenin/Tcf-dependent luciferase reporter) and FopFlash (the negative control reporter for *ß*-catenin/Tcf-binding element mutation) plasmids for 8 h followed by stimulation with S33 for 48 h, and then measured luciferase activity. The results showed that S33 upregulated TopFlash reporter activity in a dose-dependent manner without affecting the activity of the negative control reporter FopFlash (Figure 1D).

Finally, Wnt target genes were detected. The results showed that the expression of Wnt target gene *Lef1* was increased in a concentration-dependent manner, and *Axin2* also showed a significant increase after treatment with 10 μM S33 (Figure 1E).

The above results showed that S33 concentration-dependently inhibits GSK3β activity by increasing GSK3β phosphorylation level and *ß*-catenin content in the nucleus, effectively activating Wnt signaling in ST2 cells.

### S33 promotes osteogenic differentiation and proliferation of ST2 cells

**FIGURE 2:**
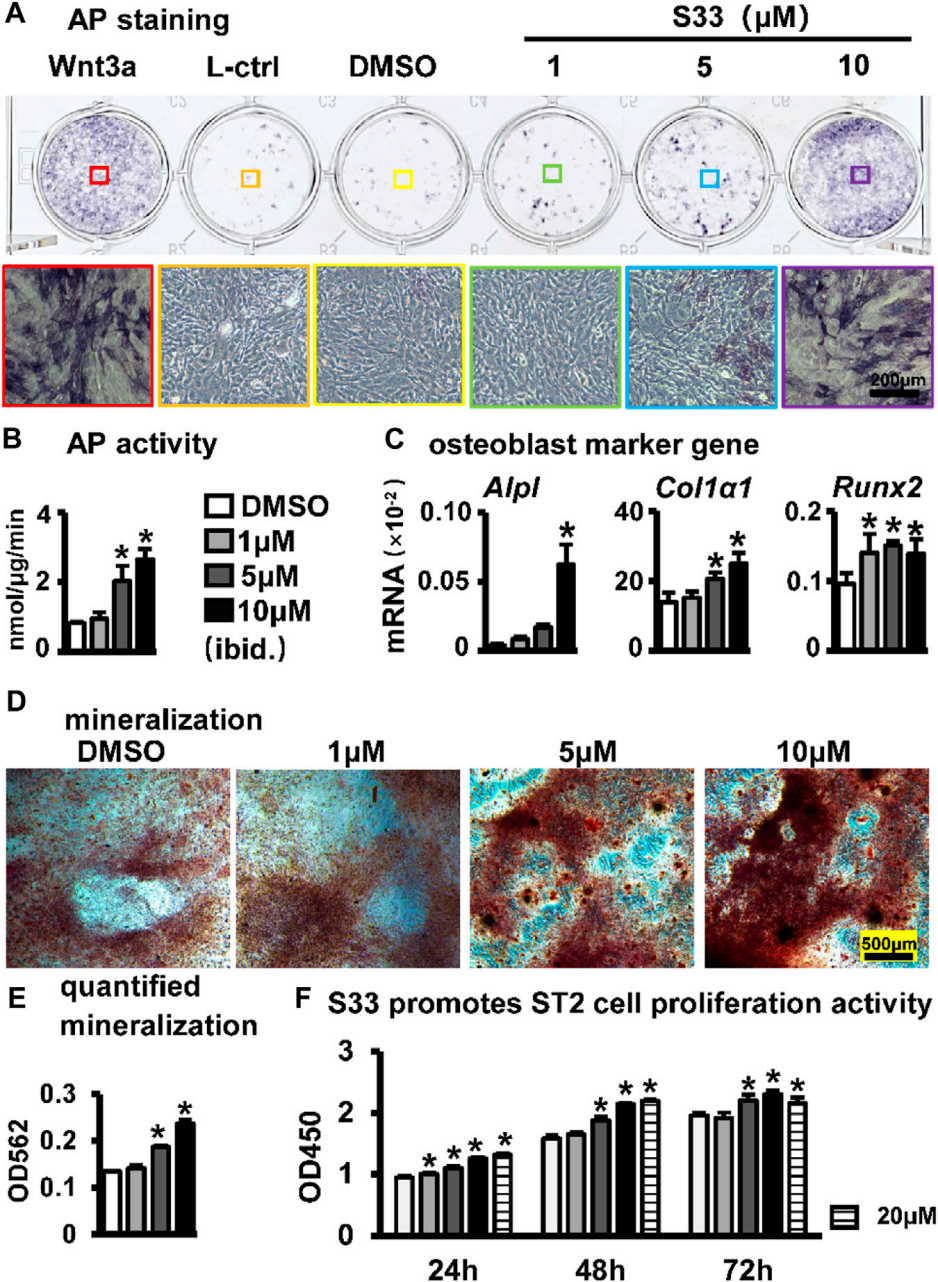
Effect of S33 on osteogenic differentiation. **(A)** ST2 cells were treated with different concentrations of S33 for 3 days, followed by AP staining. Positive and negative controls were established using Wnt3a and control L conditioned medium, respectively, scale bar = 200 µm **(B)** AP biochemical activity assay. **(C)** Expression of osteoblast marker genes **(D)** Images under the microscope of Alizarin Red S staining of bone nodules, scale bar = 500 µm. **(E)** Quantitative analysis of mineralization **(F)** CCK8 assay. *, *p* < 0.05, compared with the DMSO group, n = 3.

To assess the effect of S33 on the osteogenic differentiation of ST2 cells following the activation of canonical Wnt signaling, osteogenic differentiation analysis was performed after ST2 cells were treated with different concentrations of S33. For AP staining, conditioned media from Wnt3a and control L cells were used as positive and negative controls, respectively. Wnt3a-expressing cells were able to secrete Wnt3a protein, which promotes osteogenic differentiation of ST2 cells (Tu et al., 2007), whereas control L cells are the control of Wnt3a-expressing cells. AP staining showed that S33 increased AP expression in ST2 cells in a dose-dependent manner (Figure 2A). Subsequent quantitative AP activity assay results showed that AP activity also increased dose-dependent (Figure 2B). Meanwhile, qPCR results indicated that S33 upregulated the expression of osteoblast marker genes *Alpl*, *Col1α1*, and *Runx2* (Figure 2C).

To further assess changes in extracellular matrix (ECM) mineralization, Alizarin Red S staining and mineralization quantification were performed in ST2 cells cultured with an osteogenic medium containing different concentrations of S33 for 14 days. Similarly, S33 promoted calcium deposition in a concentration-dependent manner (Figure 2D), with mineralization quantified 1.8-fold higher than the control at 10 μM (Figure 2E).

The CCK8 assay was used to assess the effect of S33 on the proliferative activity of ST2 cells at different concentrations. After ST2 cells were treated with S33, our results showed that S33 not only had no cytotoxicity to ST2 cells in the concentration range of 0—20 μM but also promoted cell proliferation in a concentration-dependent manner (Figure 2F).

The above results showed that S33 promoted osteogenic differentiation and proliferation of ST2 cells in a concentration-dependent manner.

### Inhibition of canonical Wnt signaling reverses S33-induced increase of osteogenic differentiation in ST2 cells

**FIGURE 3:**
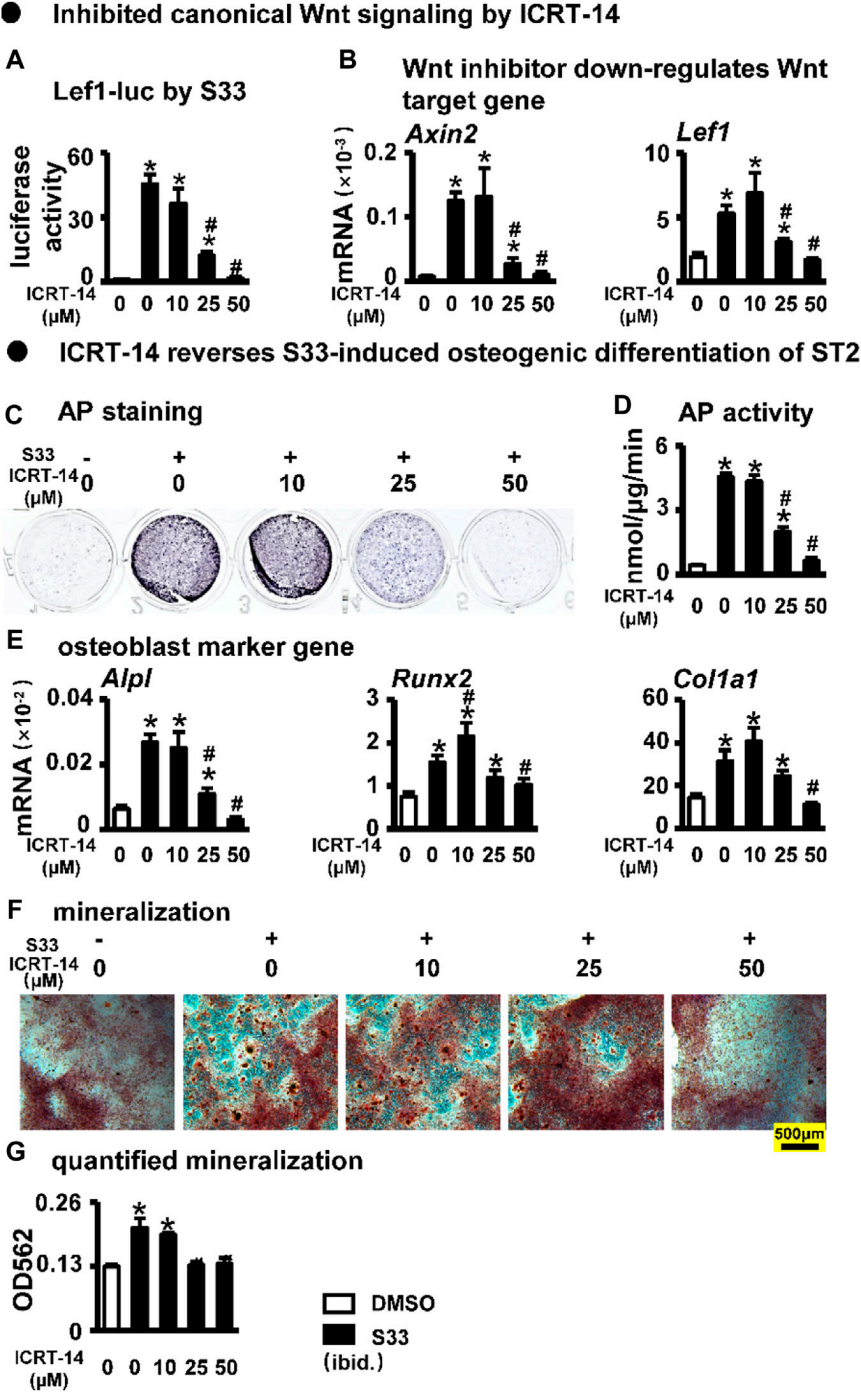
Effect of inhibiting Wnt signaling on osteogenic differentiation. **(A)** Luciferase activity. After transfection of ST2 cells with TopFlash/FopFlash plasmids, the cells were treated with S33 (10 μM) and different concentrations of inhibitors for 3 days. **(B)** Expression of Wnt target genes **(C)** AP staining. **(D)** AP biochemical activity assay **(E)** Expression of osteoblast marker genes. **(F)** Alizarin Red S staining was performed after ST2 cells were treated with ICRT-14 and S33 for 14 days and photographed under a microscope, scale bar = 500 μm, and quantification assay **(G)** was performed after this. *, *p* < 0.05, compared with the DMSO group, n = 3.

To investigate whether S33 promotes osteogenic differentiation by activating canonical Wnt signaling in ST2 cells, we used the Wnt/β-catenin/Tcf-mediated transcriptional antagonist ICRT-14 for experiments. We used different concentrations of ICRT-14 and 10 μM of S33 to act on ST2 cells for 3 days. The results showed that TopFlash reporter activity was inhibited in ICRT-14 treatment in a dose-dependent way (Figure 3A), and the expression of Wnt target genes *Axin2* and *Lef1* were significantly decreased (Figure 3B), demonstrating that Wnt signaling was effectively inhibited by ICRT-14.

We then treated ST2 cells with ICRT-14 and S33 for 3 days. AP staining and biochemical quantification showed that AP activity was significantly inhibited by ICRT-14 treatment (Figures 3C, D). qPCR results showed that the expression of osteoblast marker genes *Alpl*, *Col1α1*, and *Runx2* was decreased to DMSO levels at a concentration of 50 μM ICRT-14 (Figure 3E). Alizarin Red S staining and mineralization quantification results showed that ICRT-14 decreased the mineralization induced by S33 in ST2 cells also in a dose-dependent manner (Figures 3F, G).

These results indicate that S33 promotes osteogenic differentiation of ST2 cells by activating the canonical Wnt signaling.

### S33 inhibits adipogenic differentiation of ST2 cells

**FIGURE 4:**
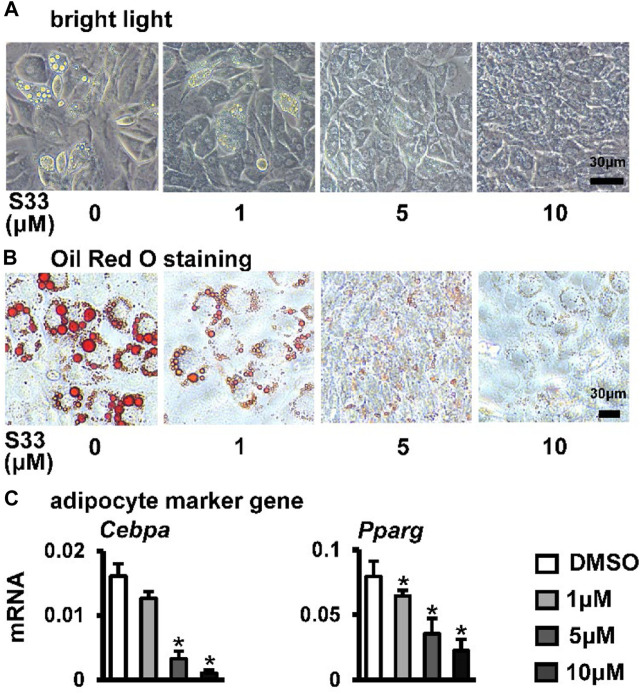
Effect of S33 on adipogenic differentiation. **(A)** Lipid droplets in ST2 cells treated with different concentrations of S33 for 7 days in the adipogenic medium under bright light, scale bar = 30 µm **(B)** Oil Red O staining images of lipid droplets in ST2 cells. Scale bar = 30 µm **(C)** qPCR detects the mRNA expression of *Pparg* and *Cebpa* after 3 days of S33 treatment. *, *p* < 0.05, compared with the DMSO group, n = 3.

Osteogenic and adipogenic differentiation are closely linked, while Wnt signaling can inhibit preadipocyte differentiation by down-regulating key adipogenic transcription factors *Cebpa*, *Pparg* (Xu et al., 2016). Therefore, we investigated whether activation of canonical Wnt signaling by S33 inhibits adipogenic differentiation. After ST2 cells were cultured with adipogenic medium for 7 days, it was observed microscopically that the cytoplasm of ST2 cells was basically occupied by lipid droplets in the DMSO group, while no significant lipid droplet was formed in the S33-treated cells (Figure 4A). And subsequent Oil Red O staining also showed a concentration-dependent reduction in lipid droplet accumulation with S33 treatment compared to the DMSO group (Figure 4B). In addition, S33 concentration-dependently inhibited the expression of *Cebpa* and *Pparg* in ST2 cells (Figure 4C).

The above results showed that S33 concentration-dependently inhibits adipogenic differentiation of ST2 cells.

### Inhibition of canonical Wnt signaling reverses S33-mediated reduction in adipogenic differentiation of ST2 cells

**FIGURE 5:**
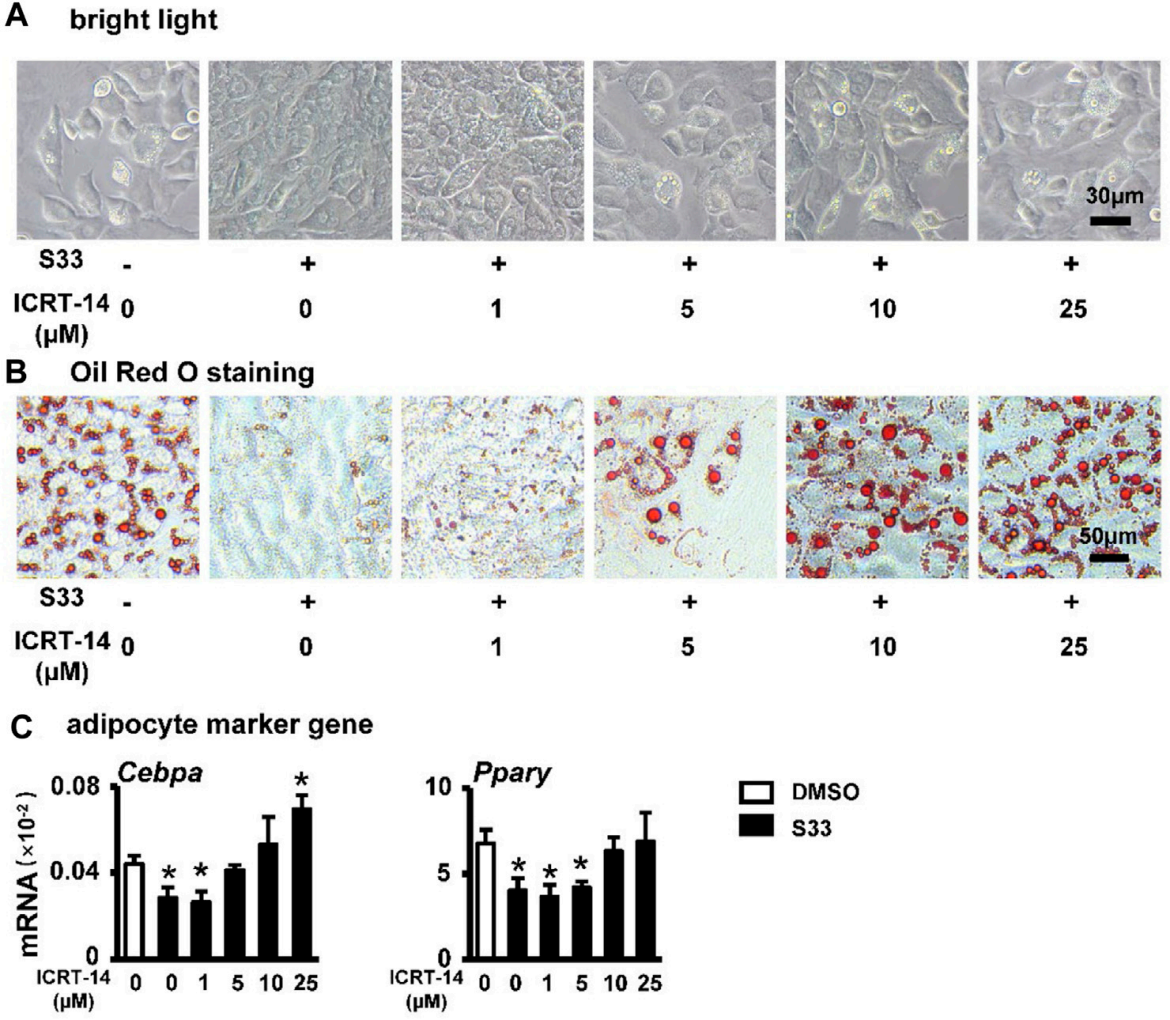
Effect of inhibiting Wnt signaling on adipogenic differentiation. **(A)** Expression of *Pparg* and *Cebpa* of ST2 cells treated with S33 and different concentrations of ICRT-14 in adipogenic medium for 3 days. To observe the changes in lipid droplet formation, ST2 cells were treated with S33 and different concentrations of ICRT-14 for 7 days in an adipogenic medium **(B)** Lipid droplets in ST2 cells under bright light, scale bar = 30 µm **(C)** Oil Red O staining images of lipid droplets in ST2 cells, scale bar = 50 μm *, *p* < 0.05, compared with the DMSO group, n = 3.

To investigate whether S33 still inhibits adipogenic differentiation by activating canonical Wnt signaling in ST2 cells, we again used different concentrations of ICRT-14 and 10 μΜ of S33 to act together in ST2 cells. After 7 days of adipogenic induction, lipid droplet accumulation in ST2 cells was reproduced with increasing concentrations of ICRT-14 (Figure 5A). This was also confirmed by Oil Red O staining. The size and density of lipid droplets in the cells basically returned to DMSO control levels after ICRT-14 concentration reached 10 μM (Figure 5B). In addition, adipogenesis-related gene assays also showed that ICRT-14 rescued S33-suppressed expression of *Cebpa* and *Pparg* (Figure 5C).

The above results clearly indicate that S33 inhibits adipogenic differentiation of ST2 cells through canonical Wnt signaling.

### S33 promotes cell proliferation in the PSCI3D scaffold

**FIGURE 6:**
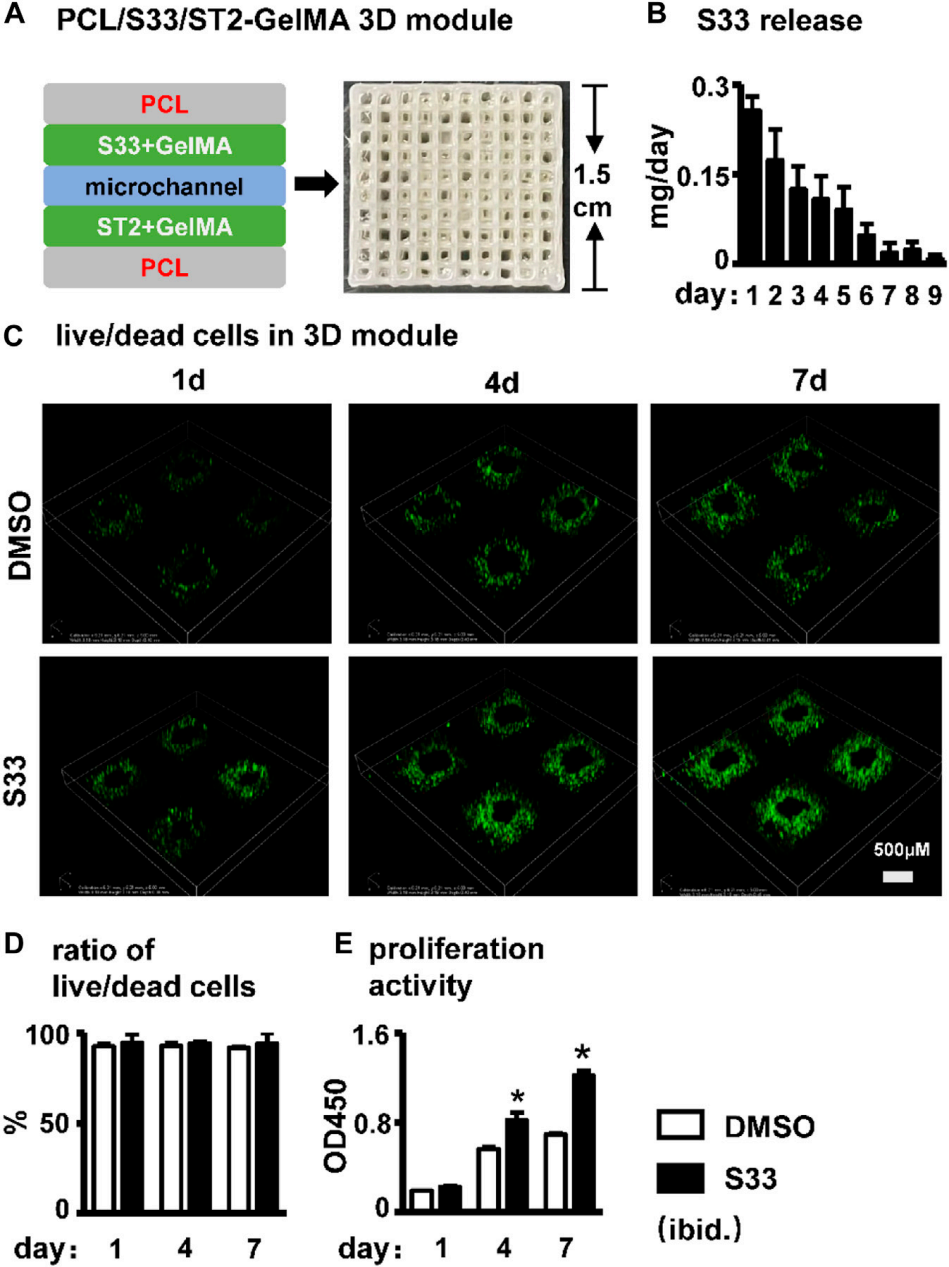
Effect of cell viability and proliferation activity in PSCI3D scaffold. **(A)** Schematic diagram of the PSCI3D scaffold and the printed scaffold **(B)** Cumulative release of S33 from PSCI3D scaffold. **(C)** Live/dead cell staining of cells at 1, 4, and 7 days in PSCI3D scaffold, scale bar = 500 μm **(D)** Live/dead cell ratio **(E)** CCK8 assay for cell proliferation activity. *, *p* < 0.05, compared with the DMSO group, n = 3.

We established PCL, S33, and cell integrated 3D printing (PSCI3D) systems (Wang P. et al., 2022) to study the function of S33. ST2 cells and S33 were mixed in GelMA hydrogel for integrated printing with PCL into a 3D scaffold for a rapid test of S33 on osteoblast differentiation (Figure 6A). The release of small molecular drugs from hydrogels plays an important role in such regard (Oliva et al., 2017), so we first test the release of S33 in the GelMA hydrogels. The experimental results showed that 25.4% of S33 was released after 1st day of culture, 81% within 7 days, and 84% within 9 days. The release content of S33 gradually decreased with time and did not show burst release (Figure 6B).

The cell survival rate of ST2 was measured by live/dead cell assay within 7 days of culture in the PSCI3D scaffold. The results showed that ST2 exhibited high cell viability in both DMSO and S33 groups within 7 days, >91% (Figures 6C, D). The cell proliferation activity assay showed that in the PSCI3D scaffold, the proliferation activity of ST2 cells in the S33 treated group increased linearly by 40% and 70% at 4 and 7 days of culture (Figure 6E).

The above results showed that S33 in the PSCI3D scaffold did not show a burst release phenomenon, and the scaffold provided a good microenvironment for cells to grow and proliferate.

### S33 promotes osteogenic differentiation and mineralization in the PSCI3D scaffold

**FIGURE 7:**
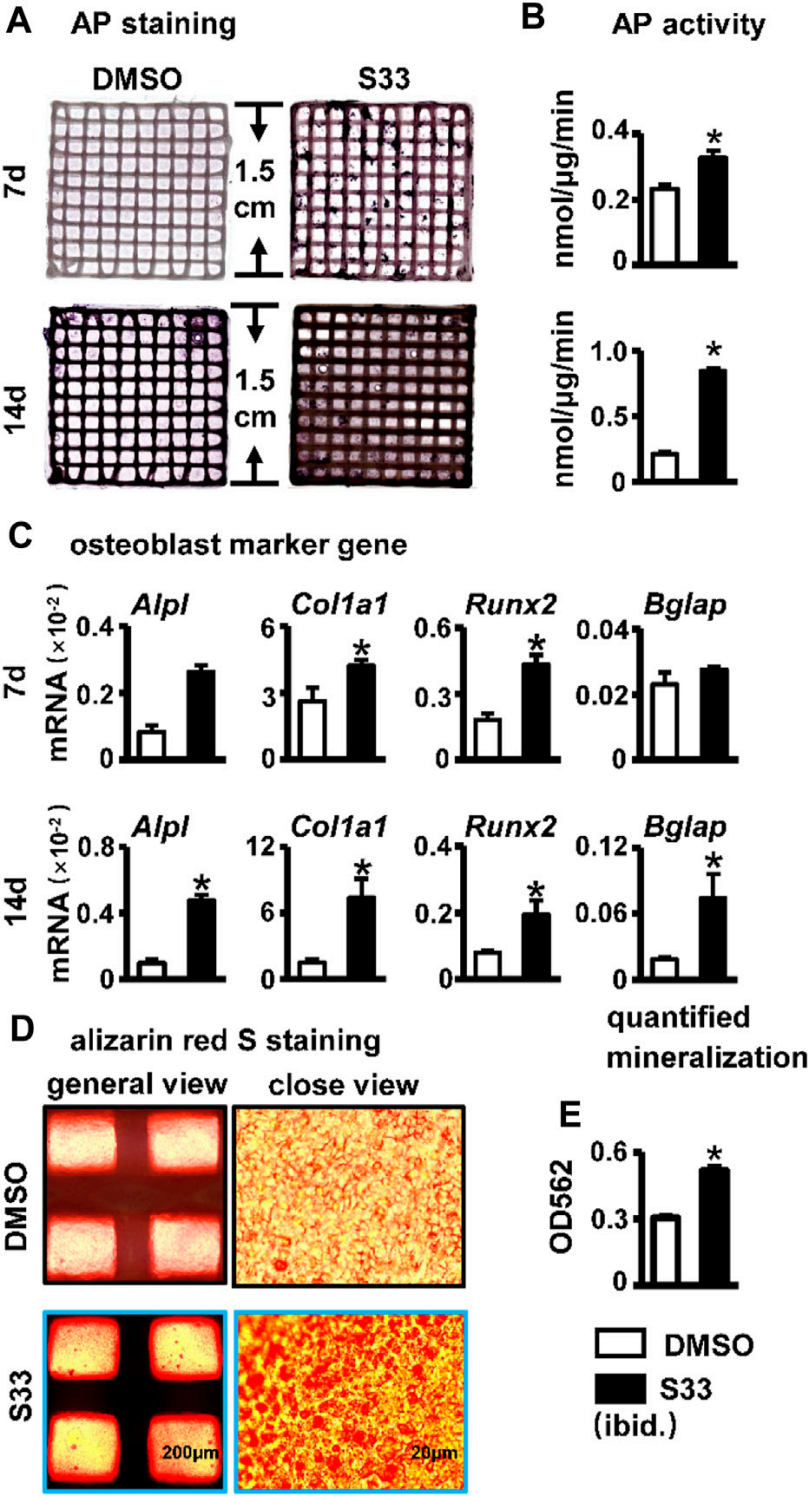
Effect of osteogenic differentiation and mineralization in PSCI3D scaffold. **(A)** AP staining of PSCI3D scaffold cultured for 7 and 14 days. **(B)** AP biochemical activity assay. **(C)** Expression of osteoblast marker genes in PSCI3D scaffold **(D)** Images under the microscope of Alizarin red S staining of bone nodules in PSCI3D scaffold, scale bars: 200 μm for the general view and 20 μm for the close view **(E)** Quantitative analysis of mineralization in PSCI3D scaffold. *, *p* < 0.05, compared with the DMSO group, n = 3.

AP staining and biochemical activity assays were performed on the PSCI3D scaffold after 7 and 14 days of culture. Compared with the DMSO group, the S33 group showed enhanced AP staining at both 7 and 14 days (Figure 7A) and increased AP activity by 1.4- and 4.0-fold, respectively (Figure 7B), demonstrating that S33 significantly promoted osteogenic differentiation in PSCI3D scaffold. This was further confirmed by qPCR in the upregulated expression of the osteoblast markers *Alpl*, *Col1a1*, and *Runx2* by S33 treatment compared to DMSO control at both 7 and 14 days, and *Bglap* expression was also increased by S33 treatment after 14 days of culture (Figure 7C).

To perform the mineralization assay, the PSCI3D scaffold was cultured in a complete medium for 7 days, followed by another 14 days in an osteogenic medium. The results of Alizarin Red S staining and quantification showed a higher number of mineral nodules in S33 treated group (Figure 7D). Additionally, mineralization was increased by approximately 1.7-fold (Figure 7E).

### S33 indirectly promotes angiogenesis

**FIGURE 8:**
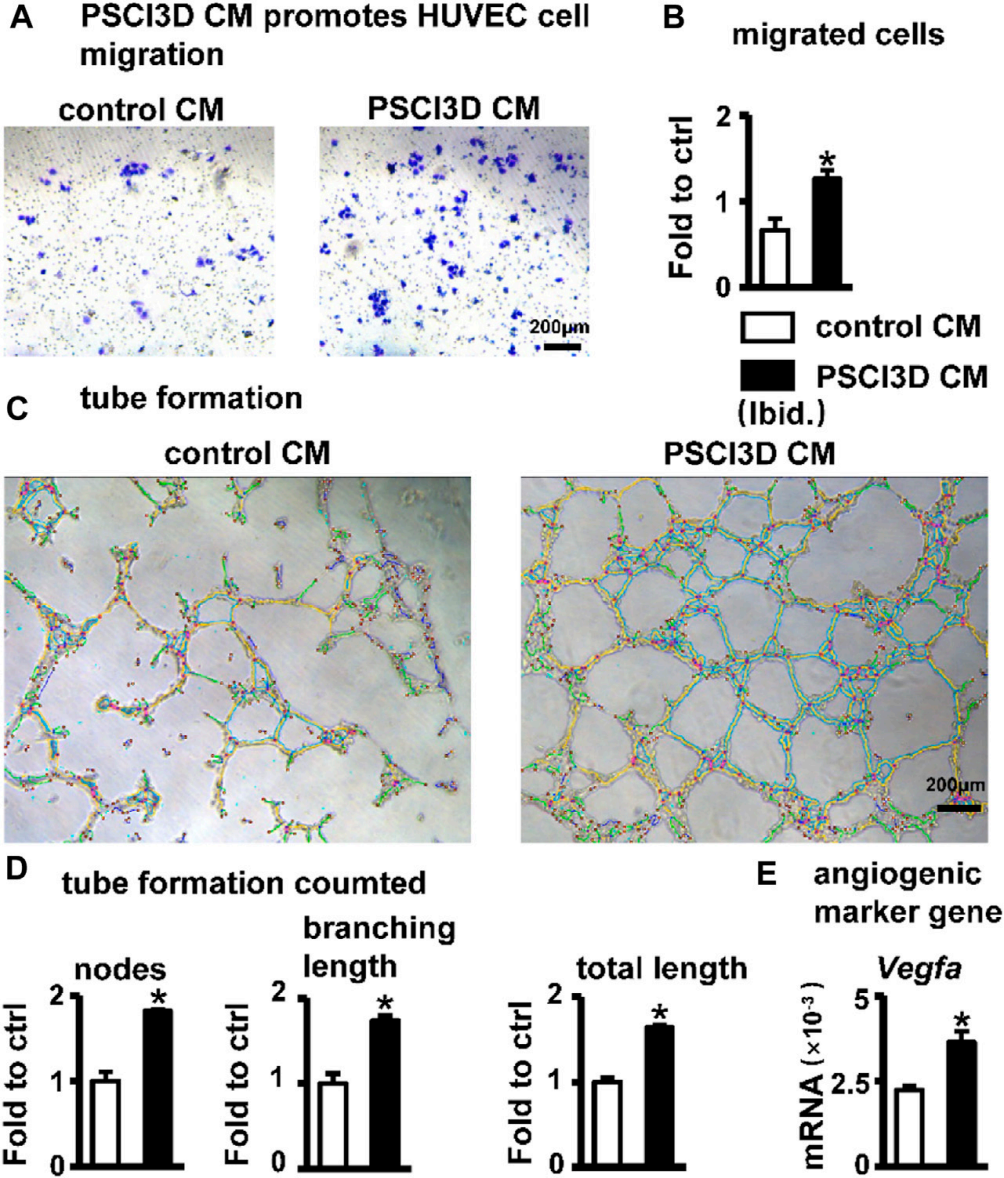
Effect of PSCI3D conditioned medium (CM) on angiogenesis in HUVEC cells. **(A)** Images of endothelial cell migration assay in the transwell chamber with conditioned medium for 48h, scale bar = 200 µm **(B)** Quantification of migrated cells. **(C)** Images of vascular tubules forming in HUVECs upon PSCI3D CM **(D)** Calculation of formed nodes, branching length, and total length **(E)** Expression of angiogenic marker *Vegfa*. *, *p* < 0.05, compared with the control CM group, n = 3.

Blood vessels are essential in bone regeneration and deliver nutrients for cell growth (Peng et al., 2020). Therefore, we examined the effect of the conditioned medium of the PSCI3D scaffold on angiogenesis. The conditioned medium of the PSCI3D scaffold facilitated the migration of HUVEC cells (Figure 8A). Migrated cells were increased by 1.9-fold compared to the controls (Figure 8B). Then, we cultured HUVEC cells in 24-well plates pre-coated with Matrigel and observed the formation of tubules after 6 h in the conditioned medium. We found that the conditioned medium increased vascular tubule formation in the PSCI3D group compared with the control medium (Figure 8C), formed nodes and branching lengths were increased by 1.82 and 1.73-fold, respectively, and total lengths by 1.64-fold compared to the control group (Figure 8D). The angiogenic process is heavily influenced by the significant role played by the vascular endothelial growth factor *Vegfa* (Sun, 2012), and qPCR results showed increased *Vegfa* expression in ST2 cells in the PSCI3D scaffold (Figure 8E).

The above results showed that S33 may have a pro-angiogenic effect when acting *in vivo*.

## Discussion

In this study, we investigated the osteogenic effect of GSK3β inhibitor S33. We utilized S33 to inhibit GSK3β kinase activity and confirmed that S33 is a potent Wnt agonist in murine bone marrow stromal cell ST2. We then show that S33 specifically stimulates osteogenesis and inhibits adipogenesis by downregulating *Pparg* and *Cebpa* expression via canonical Wnt signaling. Inhibition of Wnt signaling by the Wnt transcriptional inhibitor ICRT-14 disrupts the bone-promoting and adipogenesis-inhibiting effects of S33, respectively. In view of the characteristics of increased adipogenesis and decreased osteogenesis of BMSCs in elderly patients with osteoporosis, S33 may have a potential therapeutic effect.

The effect of S33 in promoting osteogenic differentiation was also reproduced in MC3T3-E1 cells (Supplementary Figures S1A–E). Because MC3T3-E1 cells are pre-osteoblasts and have exceeded the common cellular stage of osteogenesis and adipogenesis in the development of osteoblasts, they cannot be induced into adipocytes. Therefore, the inhibitory effect of S33 on adipogenic differentiation has not been detected in the MC3T3-E1 stage.

We printed the PSCI3D functional scaffold using a newly developed PCL and cell integrated 3D bioprinting technology to simulate the developmental microenvironment *in vivo*. In the PSCI3D scaffold, the survival rate of cells reached 94% within 7 days, so ST2 cells grew linearly and greatly increased cell proliferation activity. High proliferation activity and survival rate guarantee subsequent osteogenic differentiation and mineralization. And we also found that the conditioned medium of the PSCI3D scaffold could effectively promote the migration and tubule formation of HUVEC cells, indicating that the PSCI3D scaffold enhances angiogenesis, which is essential for bone formation.

S33 has a variety of biological functions and may have translational application value in the treatment of osteoporosis. At present, PTH, PTHrP, and sclerostin antibodies for the treatment of osteoporosis are all protein drugs that have defects such as immunogenicity, easy degradation, high price, short half-life, and poor permeability (Vargason et al., 2021). These side effects limit their use to some extent. However, small molecule drugs have been a research hotspot due to their good stability, solubility, easier processing, and low cost (Vargason et al., 2021). Like anti-sclerostin antibodies, the small molecule S33 acts through the Wnt/β-catenin pathway to promote osteogenic differentiation, in addition to inhibiting adipogenic differentiation, promoting angiogenesis, and has the potential to inhibit osteoclast differentiation. We found that S33 increased *Opg* expression in ST2 cells and decreased the *RANKL/Opg* ratio, potentially inhibiting osteoclast differentiation (Supplementary Figure S3).

S33 is an effective and safe osteogenic drug. The Canonical Wnt pathway is important in cell proliferation, differentiation, polarization, and migration (Wodarz and Nusse, 1998). Therefore, the safety of drugs targeting this pathway is very important when developing drugs (Kahn, 2014). In the PSCI3D scaffold, the sustained-release system released 25.6% of S33 on the first day and slowly released 81.7% within 7 days. At 7 days, S33 promoted osteoblast differentiation, which increased by 1.4-fold compared to the control group, but at 14 days, osteoblast differentiation increased even higher, reaching 4.0-fold. Although the concentration of S33 is very low at 7 days, the affected ST2 cells can continue to proliferate and differentiate. Thus, the drug’s effect is limited to the local area and does not affect the tissues and cells around the scaffold. In addition, the conditioned medium of the PSCI3D scaffold promotes angiogenesis (Figures 8A–E). This indicates that the released S33 forms a developmental microenvironment guiding cells to proliferate and differentiate into osteoblasts. After slowly releasing most of S33 within 7 days, it can still maintain the osteogenic differentiation of the cells, and the microenvironment may secrete angiogenic factors such as *Vegfa* for angiogenesis. Therefore, the development microenvironment formed in the PSCI3D scaffold promotes osteogenic differentiation and angiogenesis.

Another important innovation in this study is the preparation of the PSCI3D scaffold by alternately printing strips of PCL, cell-laden hydrogel, and S33-laden hydrogel through a newly developed integrated 3D bioprinting technology, which creates an osteogenic niche. Printing hard materials together with cells has been a huge challenge because the problem of low cell survival rate cannot be well solved, making cells unable to differentiate subsequently (Valot et al., 2019). In 2016, Kang et al. established an ITOP to print PCL and stem cell scaffold that can induce bone vascularization after implantation *in vivo* (Kang et al., 2016), becoming a milestone in 3D bioprinting technology. However, in the following period, 3D bioprinting technology was still limited by the lack of a developmentally functional microenvironment and failed to make a further breakthrough.

As we previously reported (Wang P. et al., 2022), we reserved space for cells to exchange substances by alternately printing strips of PCL, cell-laden hydrogel, and S33-laden hydrogel. This resulted in a 45.3% and 75.4% increase in cell proliferation activity at 4 days and 7 days of cultivation in a 3D environment, which was a little greater than that in a two-dimensional environment. And we also provided mechanical support for cell adhesion and proliferation through blue light-curable GelMA hydrogel. These methods have greatly improved the survival rate of cells, which can remain above 94% within 7 days, slightly higher than that in 2D culture (unpublished data). In addition, S33 in the hydrogel can still function for a long time after releasing most of the S33 at 7 days, well mimicking the effect of S33 on cells *in vivo* in a three-dimensional environment. Therefore, our reconstructed PSCI3D scaffold can simulate the developmental microenvironment *in vivo* and rapidly verify the effect of S33 on cell differentiation *in vivo*, which also provides a possible method for the rapid identification of the *in vivo* effect of drugs in the future.

Another interesting function of S33’s ability is to promote angiogenesis. We found that PSCI3D conditioned medium exhibits a significant angiogenic effect (Figures 8A–E). To exclude the impact of residual S33 in a conditioned medium, we used S33 to directly stimulate HUVECs, but unfortunately, no angiogenesis function was found (Supplementary Figures S2A–C). However, our previous work reveals that osteocyte with SKL2001-activated Wnt signaling promotes angiogenesis (Liu et al., 2022). Thus, similar to the previous mode, S33 may indirectly promote angiogenesis by stimulating ST2 cells to release angiogenic factors such as *Vegfa* (Figure 8E), but the specific mechanism needs further investigation.

## Conclusion

All in all, we found that SB216763, a highly specific GSK3β inhibitor has multiple functions such as osteogenic and anti-adipogenic effects and angiogenic potential, and S33 may also have another potential to inhibit osteoclastogenesis. These features make S33 a good candidate drug for therapeutics against elderly osteoporosis. Amazingly, when S33 is almost released in the PSCI3D scaffold at 7 days of cultivation, it creates an osteogenic microenvironment to produce better effects of osteoblast differentiation and mineralization. For the time being, we call this drug sustained-release in the 3D scaffold creating an osteogenic niche that continues to play a role in bone formation. The PSCI3D scaffold can be used as a bioactive material to bionically reconstruct artificial bone organs.

## Data Availability

The datasets presented in this study can be found in online repositories. The names of the repository/repositories and accession number(s) can be found in the article/Supplementary Material.
