# Supplementary material for: Sustained release of a highly specific GSK3β inhibitor SB216763 in the PCL scaffold creates an osteogenic niche for osteogenesis, anti-adipogenesis, and potential angiogenesis
Source: Front Bioeng Biotechnol. 2023 Jul 28;11:1215233. doi: 10.3389/fbioe.2023.1215233 (PMC10419179; doi:10.3389/fbioe.2023.1215233)
Supplement: Supplementary file 1 [file DataSheet1.docx]

Supplementary Material

Sustained release of a highly specific GSK3β inhibitor SB216763 in the PCL scaffold creates an osteogenic niche for osteogenesis, anti-adipogenesis, and potential angiogenesis

Weimin Gong^1†^, Molin Li^1†^, Lizhou Zhao^1†^, Pengtao Wang^1^, Xiaofang Wang^1^, Bo Wang^1^, Xing Liu^2*^, Xiaolin Tu^1*^

*** Correspondence:**Xiaolin Tu
[xtu@cqmu.edu.cn](mailto:xtu@cqmu.edu.cn)

Xing Liu
liuxingda@126.com

**^†^** These authors contributed equally to this work

# Supplementary Figures and Tables

## Supplementary Figures

**
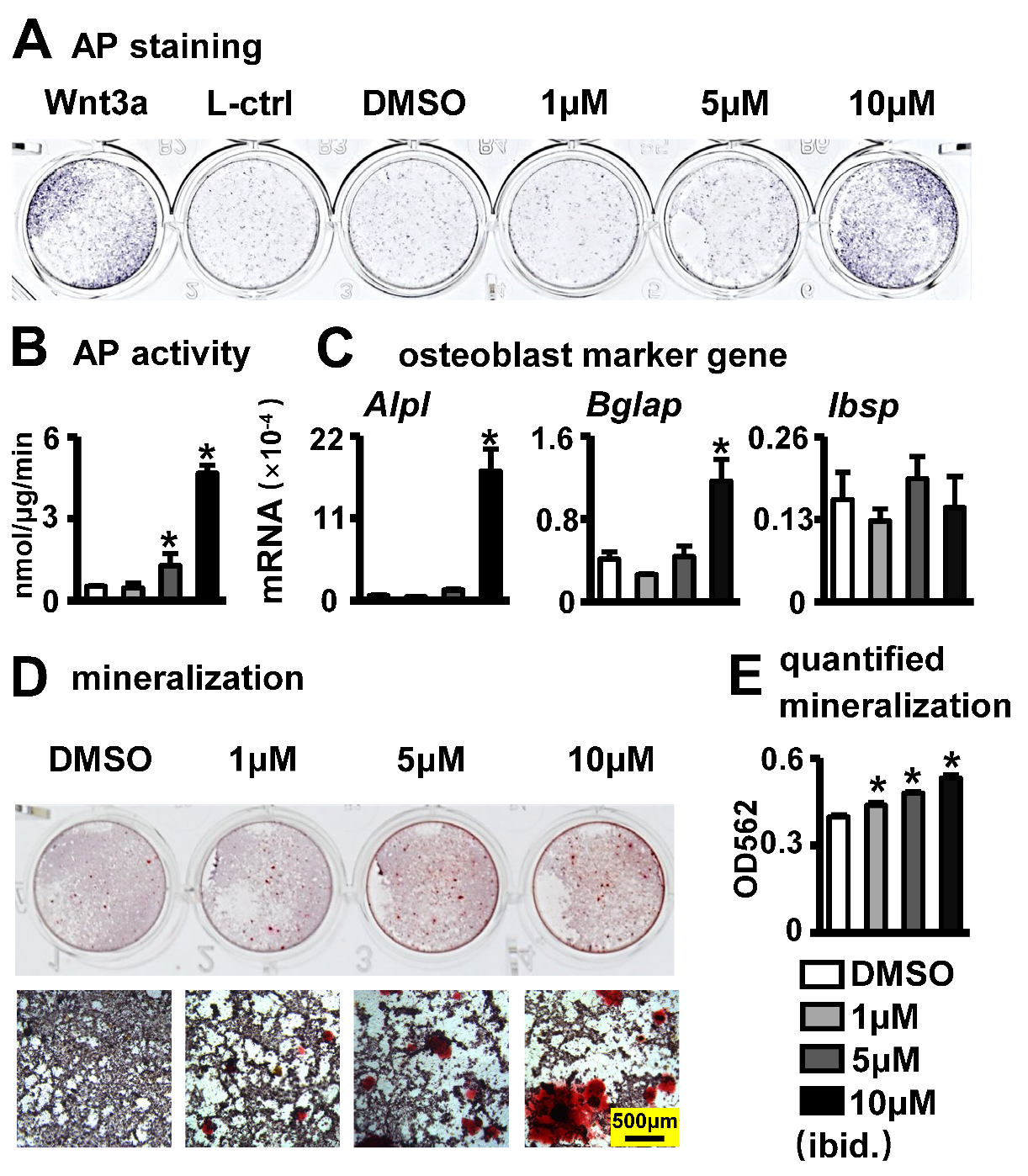
**

**Supplementary Figure 1.** Effect of S33 on osteogenic differentiation in MC3T3-E1 cells. MC3T3-E1 was treated with different concentrations of S33 for 7 days. (A) AP staining of MC3T3-E1 cells, in which Wnt3a and L control conditioned medium were used as positive and negative controls, respectively. (B) AP biochemical activity assay. (C) Expression of osteoblast marker genes. (D) Alizarin Red S staining of formed bone nodules of MC3T3-E1 cells treated with different concentrations of S33 in osteogenic medium for 14 days, scale bar = 500 µm. (E) Quantification assay of mineralization. *, p < 0.05, compared with the DMSO group, n = 3.


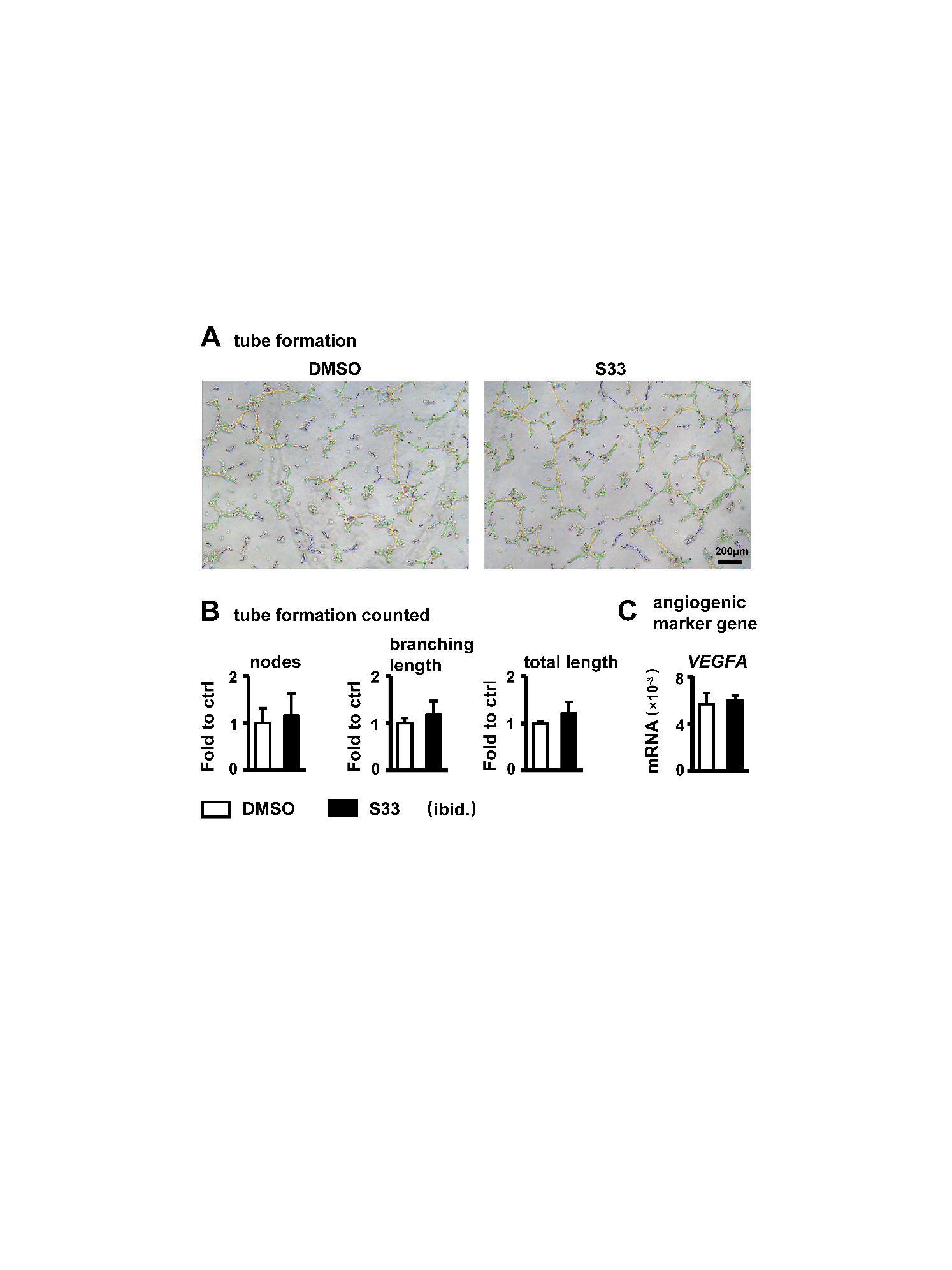


**Supplementary Figure 2.** Effect of S33 on angiogenesis in HUVECs. (A) Images of vascular tubules forming in HUVECs upon different concentrations of S33, scale bar = 200 µm. (B) Calculation of formed nodes, branching length, and total length. (C) Expression of angiogenic gene VEGFA. *, p < 0.05, compared with the DMSO group, n = 3.





**Supplementary Figure 3.** Potential effects of S33 on osteoclast differentiation. ST2 cells were treated with different concentrations of S33 for 3 days. (A) qPCR was used to detect the osteoclast cytokines expression. (B) The ratio of RANKL/Opg. *, p < 0.05, compared with the DMSO group, n = 3.
